# Editor-in-Chief changes at *Health Policy and Planning*

**DOI:** 10.1093/heapol/czae037

**Published:** 2024-06-25

**Authors:** Sandra Mounier-Jack, Virginia Wiseman, Lucy Gilson

**Affiliations:** Department of Global Health and Development, London School of Hygiene and Tropical Medicine, UK; UNICEF HQ Immunization Unit, New York; Department of Global Health and Development, London School of Hygiene and Tropical Medicine, UK; Chair of Health Economics & Health Systems, Kirby Institute, University of New South Wales, Australia; Department of Global Health and Development, London School of Hygiene and Tropical Medicine, UK; School of Public Health, University of Cape Town, South Africa

We would like to share the news of the stepping down of our wonderful co-Editor-in-Chief, Professor Sandra Mounier-Jack, from our journal *Health Policy and Planning*. Sandra has been a highly valued co-Editor-in-Chief for 11 years and her work with the journal has been transformative. This has included her work on passionately expanding our editorial team, working tirelessly on numerous high-impact journal supplement publications, and most recently supporting our transition from a hybrid to a fully open access journal.

Sandra is professor of Health Systems and Policy at the London School of Hygiene & Tropical Medicine and has many incredible scientific and academic accomplishments including extensive experience in analysing and strengthening immunization programmes in low, middle and high countries. We thank her for her time, expertise and hard work in co-running the journal and wish her the best of luck at the UNICEF HQ Immunization Unit in New York.

With that news shared, we are excited to announce that Professor Lucy Gilson has now joined the leadership team of *Health Policy and Planning* as co-Editor-in-Chief to work alongside our current Editor, Professor Virginia Wiseman.

Professor Lucy Gilson has long-been linked to *Health Policy and Planning’s* editorial team including most recently, as Section Editor for Health Policy Processes. She is very well placed to take over this important role for our journal with her strong leadership in the field of health policy and health systems research, as well as her dedication to mentoring and supporting early career researchers and editors, especially those in Africa.

Lucy Gilson holds the appointment of professor both at the University of Cape Town and the London School of Hygiene and Tropical Medicine, UK, and is an honorary professor at the University of the Western Cape.

Throughout her career, her interdisciplinary research has been driven by a concern for equity in health and health care. This has involved conceptual and empirical work on issues of health system organization and governance, leadership and management, health care financing and health policy change. She has conducted a wide range of collaborative research with colleagues in Eastern and Southern Africa, South Africa and in Asia. Lucy has also played a leading role in capacity strengthening for health policy and systems research in general, and health policy analysis more specifically.

So while we sadly say farewell to Sandra who has laid so many important foundations at *Health Policy and Planning* that have enabled us to grow and adapt over the past 15 years, it is with much excitement that we welcome Lucy onboard to help guide us into a new era of academic publishing. Thank you both!

From all the team at *Health Policy and Planning*.

